# The Surgical Correction of Urogenital Sinus in Patients with DSD: 15 Years after Description of Total Urogenital Mobilization in Children

**DOI:** 10.3389/fped.2013.00041

**Published:** 2013-11-21

**Authors:** Barbara M. Ludwikowski, Ricardo González

**Affiliations:** ^1^Auf der Bult Kinder und Jugendkrankenhaus, Hannover, Germany; ^2^Charité Universitätsmedizin Berlin, Virchow Klinikum, Berlin, Germany

**Keywords:** urogenital sinus, disorders of sex development, congenital adrenal hyperplasia, cloaca, feminizing genitoplasty

## Abstract

Total urogenital sinus mobilization has been applied to the surgical correction of virilized females and has mostly replaced older techniques. Concerns have been raised about the effect of this operation on urinary continence. Here we review the literature on this topic since the description of the technique 15 years ago. Technical aspects and correct nomenclature are discussed. We emphasize that the term “total” refers to an en-bloc dissection and not to the extent of the proximal dissection. No cases of urinary incontinence have been reported following this operation. It is yet too early to evaluate results regarding sexual function but it is likely that the use of a posterior skin flap to augment the introitus will minimize the development of introital stenosis.

Persistence of the urogenital sinus (UGS) is a common feature of a variety of congenital anomalies, among them: (1) XX DSD, namely females exposed to androgens in fetal life; (2) as an isolated malformation unrelated to masculinization or rectal malformation and; (3) in persistence of the cloaca. Although there are important anatomical and functional differences among the different types of UGS the surgical techniques used for its correction bear similarities.

The goal of the surgical correction of UGS is the creation of separate openings in the vulva for the urethra and the vagina with preservation of the function of both organs. Early surgical techniques involved complete or partial separation of the urethra from the vagina, at times preserving the sinus as the urethra and doing either a vaginal “pull through” (1) or creating skin or mucosal flaps to construct the distal vagina (2, 3). These operations are often tedious and fraught with complications including vesico-urethral fistulas, urethral and vaginal stenosis (4), complete vaginal occlusion, and urinary incontinence (5).

In 1997, Peña published a new technique for repair of the UGS in girls with persistence of the cloaca which avoided the separation of the urethra from the vagina. The technique involved the en-bloc mobilization of both structures to bring them down to the perineum creating separate openings. This maneuver saved time and reduced the number of complications in the eight children described and was called total urogenital mobilization (TUM) (5). By necessity, in cases of cloaca, the approach was posterior sagittal. Two years later Ludwikowski et al. (6) described the application of this technique not only to children with persistence of the cloaca but also to children with congenital adrenal hyperplasia (CAH) and primary isolated UGS among others. One innovation introduced in that publication was that in children with normal anus, the approach to the TUM was perineal. This publication was followed 2 years later by another that included six patients with normal anus and rectum in whom the procedure was done exclusively through the perineum.

Subsequently, some authors expressed concerns that TUM may compromise urinary continence and bladder function (7) and a procedure named “partial mobilization of the UGS” (PUM) was described (8). The authors of this report incorrectly interpreted that the term total referred to the extent of proximal dissection used for the mobilization rather than to the concept of en-bloc dissection of the urethra and vagina (9). A more correct terminology is TUM with proximal or distal dissection; therefore these terms will be used in this article.

Many reports on the outcome of TUM include patients with a variety of diagnoses which tends to make interpretation of results difficult.

Here we review the published results of TUM in patients with XX DSD without associated anorectal malformations.

## Methods

Pubmed search under the headings: “UGS, vaginoplasty, feminizing genitoplasty, and TUM” was conducted. After collecting all abstracts, full length articles of those which included UGS mobilization in DSD patients were reviewed. Results regarding number of patients, diagnosis, urinary continence, and potential vaginal stenosis were recorded.

## Results

The persistence of the UGS in cases of genetic females exposed to androgens in the first few weeks of embryogenesis is almost universal. In virilized XX individuals the urethra and vagina share a common opening but the urethra and vagina develop normally proximal to the persistent UGS. Therefore, the urethra proximal to the confluence is of normal length (10). The goal of surgical correction is to create separate urethral and vaginal openings in the vulva preserving the function of both organs. The surgical reconstruction of these malformations also includes a vulvoplasty and sometimes a clitoroplasty. The timing and techniques to achieve these goals has been the object of much controversy and are outside the scope of this review (11). We shall limit the discussion to the reported outcomes for the correction of the UGS in XX DSD.

Following the description of the technique (6), Kryger and González studied 13 girls after TUM with non-invasive urodynamics and a questionnaire and found no cases of incontinence attributable to the operation (12). Farkas et al. (13) reported results of en-bloc UGS mobilization in 46 patients with a mean follow-up of 4.7 years and found no instances of vaginal stenosis of incontinence, however their report focuses mostly on the surgical technique and details of how the postoperative evaluation was perform are scant. Braga et al. reported on 10 girls with CAH followed for 26 months after distal TUM and saw no evidence of incontinence or vaginal stenosis. In cases with “high confluence,” they conducted a limited separation of the urethra from the vagina (14). Similar to Ludwikowski et al. (6), they used a posterior skin flap to augment the circumference of the vaginal introitus and to avoid a circumferential suture line. Camanni et al. followed six girls with CAH corrected by TUM and found no instances of bladder dysfunction or incontinence (15). In prospective report by Braga et al. (16) of 24 girls with CAH operated at a mean age of 28 months and a mean follow-up of 25 months, no cases of urinary incontinence or vaginal stenosis were encountered. These authors stopped the anterior dissection at the pubourethral ligaments (distal TUM) and were successful in obtaining sufficient mobilization in 96% of the patients. The authors also use the term “partial mobilization” referring to distal mobilization but they did an en-bloc dissection.

More recently, Palmer et al. reported on the continence status of a population with mixed diagnoses which included CAH, cloaca, and primary UGS (17). Among the seven patients with CAH there were no cases of urinary incontinence and the authors found no difference in results between proximal and distal dissection.

The results of the literature search are presented in Table 1. In summary, all series of XX DSD treated with TUM since its description in 1999 point out to the advantages of this approach. No patient primarily operated with the classical TUM has been reported to have urinary incontinence. Data to evaluate the adequacy of the vagina for sexual intercourse have not yet been reported and need to wait long term follow-up studies.

**Table 1:** **Summary of published results regarding urinary continence after TUM**.

Reference	Diagnosis	Number ofcases	Follow-up(mean – months)	Technique	Incontinence	Vaginalstenosis
Kryger and González (12)	DSD (9 CAH)	13	40	TUM	0	N/A
Camanni et al. (15)	CAH	6	60	TUM	0	N/A
Braga et al. (14)	CAH	24	25	TUM (distal)	0	0
Farkas et al. (13)	DSD (44 CAH)	46	56	TUM (distal)	0	0
Palmer et al. (17)	Mixed (7 CAH)	25	51	TUM (proximal and distal)	0	N/A
Total		114			0

## Discussion

We analyzed the results of TUM performed to correct UGS in virilized females. Although many series in the literature include cases of CAH, cloaca and primary UGS, and other forms of DSD, it should be recognized that the variable anatomy and possible associated neurological anomalies may have a profound influence in urinary continence independent from the operation performed (18).

**Figure 1:**
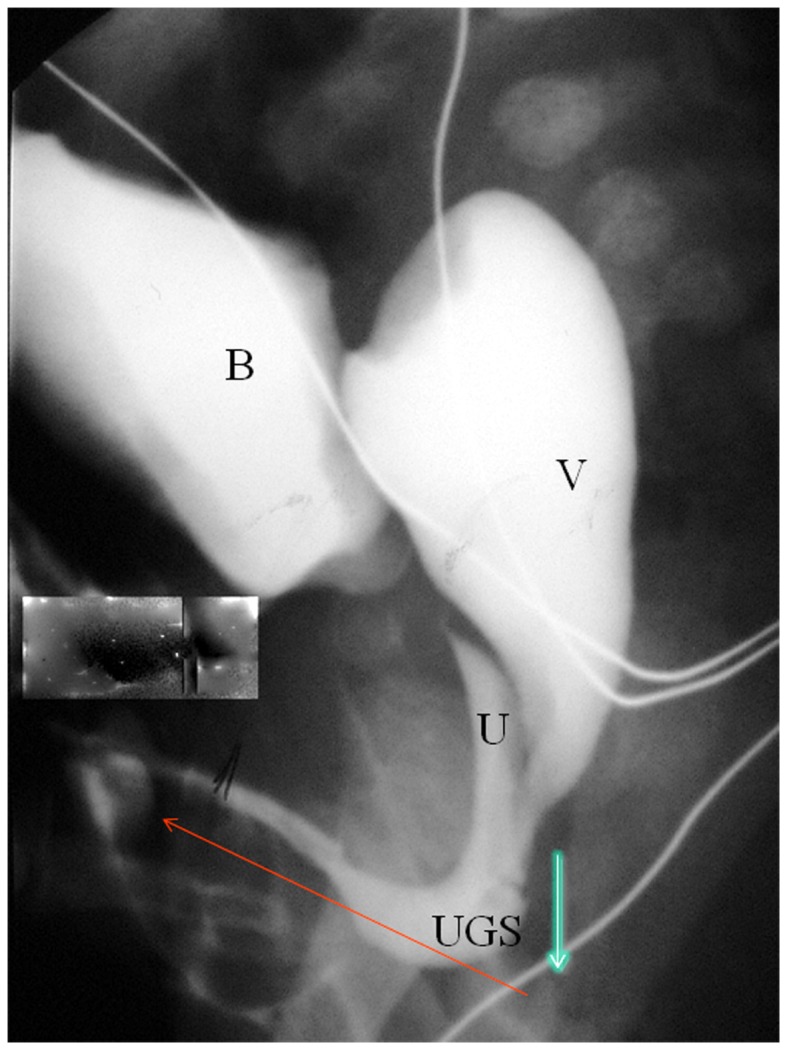
**Contrast study in patient with typical anatomy in a Prader 3–5 CAH**. B, bladder; U, urethra; V, vagina. The blue arrow indicates the distance between the confluence and the perineal skin. The red arrow indicates the length of the UGS which is not relevant to the difficulty of the mobilization. The excess tissue can be used to create a mucosa lining of the vestibule during vulvoplasty. (6). Because of this, we no longer restrict TUM to UGS less than 3 cm in length.

**Figure 2:**
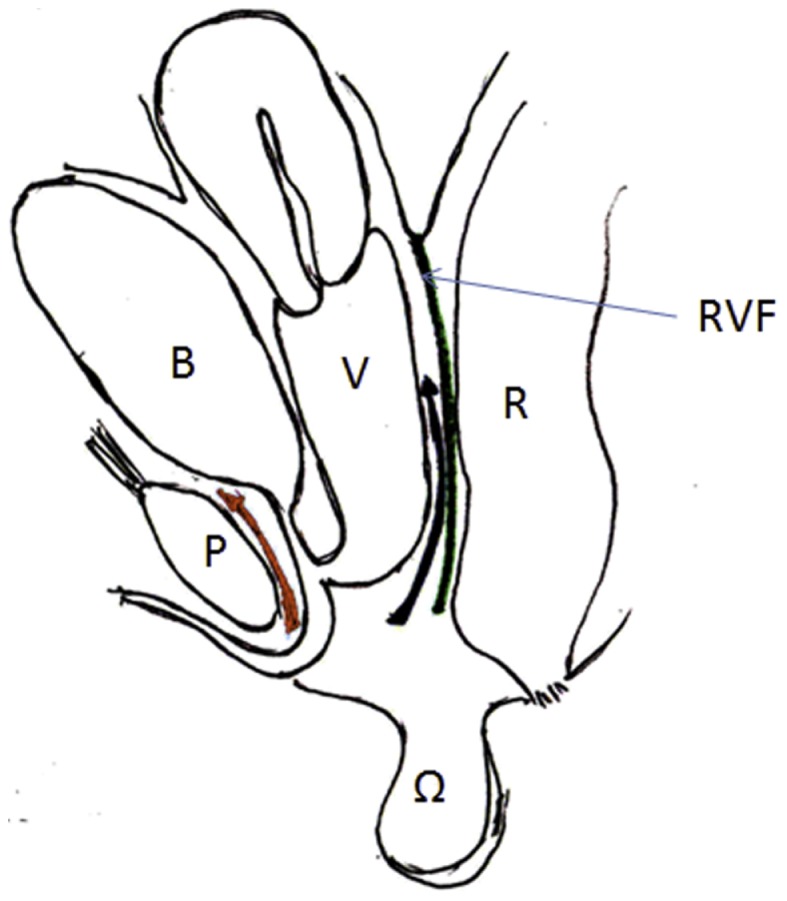
**Artist’s representation of the anatomy and planes of dissection**. B, bladder; U, urethra; V, vagina; P, pubis; RVF, rectovaginal fascia. Anteriorly the dissection should be close to the pubis, posteriorly close to the vagina, and laterally a few millimeters away from the vaginal wall.

The application of TUM to cases of virilized females has largely replaced the use of other procedures that required the separation of the urethra from the vagina. The advantages of this technique are clear; avoiding separation of the urethra from the vagina reduces surgical time and complications and may avoid potential damage to urethral innervation. Many authors mention the length of the UGS as a criterion to select the surgical technique. In fact, the length of the sinus may be irrelevant since much of its course is in the perineum, parallel to the surface. More important to predict the easy or complexity of the surgery is the distance from the confluence of the vagina and urethra to the perineal skin (Figure 1). We routinely perform endoscopy at the time of surgery. In cases of virilized XX females (CAH, aromatase deficiency, prenatal exposure to androgens) the urethra above the confluence is normal. Whether the anterior dissection to mobilize the UGS stops at the pubourethral al ligaments or extends to the Retzius space, depends entirely on the needs in a particular case and is determined by the anatomical situation encountered. In any event the anatomy of the pubourethral ligaments in nulliparous females is variable (19) and proponents of limited anterior dissection (erroneously called partial UGS mobilization or PUM) do not clearly indicate whether the stop the dissection at the distal, intermediate, or proximal ligaments. For this reason, whenever-bloc dissection of the urethra and vagina is done the procedure should be named TUM which may be qualified as distal or proximal according to how far the anterior dissection was carried. Based on anatomical studies, Kalfa et al. recommended not extending the anterior dissection above the upper limit of the pubis. They also saw rich innervation along the lateral aspects of the vagina (20). Based on this study, it makes sense to keep the anterior dissection close to the pubic bone and the lateral dissection a few millimeters away from the vagina (20) (Figure 2).

Posteriorly, the dissection should always be carried to the peritoneal reflexion to facilitate the descent of the posterior vaginal wall and the suture of the Ω flap. It is important to keep the posterior dissection close to the vagina in order to avoid disruption of the rectovaginal fascia which seems important for normal rectal function (21).

We operate all those cases perineally in the dorsal lithotomy position. We see no advantage in the prone position which makes the vulvoplasty difficult. Furthermore, in cases of primary XX DSD, the use of the transanorectal approach is overly aggressive, hinders the use of a posterior flap and increases the potential for serious complications (22, 23).

In short, data published since the description of TUM fail to demonstrate any adverse effect of the operation on voiding or continence.

Interestingly, Davies et al. investigated 19 adult women with CAH of whom 16 had had corrective surgery in childhood. They found an incidence of urge incontinence of 68% and stress urinary incontinence (SUI) of 47%, significantly greater than in controls (24). It is not possible to determine whether the incontinence was due to the previous surgery (likely to involve techniques no longer in use) or CAH.

**Figure 3:**
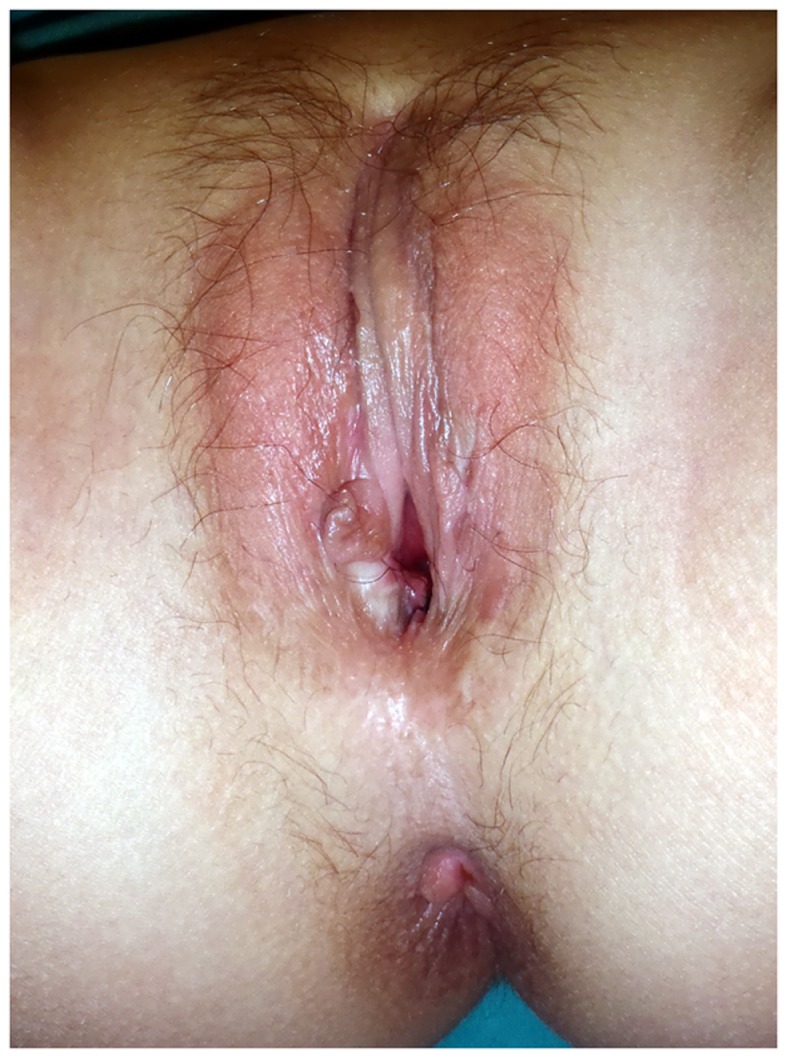
**Appearance of the vulva 6 years after TUM with posterior Ω flap**. The patient was virilized (Prader 4) because of maternal exposure to androgens.

Most authors have combined TUM with a posterior flap of perineal skin to augment the circumference of the suture line and prevent introital stenosis (6, 25). The use of an omega shaped flap improves the appearance of the posterior fourchette (12, 26) (Figure 3).

Gosalbez et al. (27) proposed abandoning the use of the posterior flap to improve cosmesis. Unfortunately, their report includes a mixture of cases of DSD and cloaca and the follow was too short to make their recommendations valid. Using older surgical techniques, Bailez et al. reported a high incidence of vaginal stenosis after reconstruction in infancy (4). However it is not clear what techniques were used or if a posterior flap was used.

Passerini-Glazel also avoided separation of the urethra from the vagina but instead of mobilizing the UGS sinus en-bloc his technique involved anastomosing the inverted distal UGS to the distal end of the vagina left in its original position (3). Lesma et al. (28) followed 46 of 82 females operated with this technique and found that adequate vaginal caliber was achieved in 20 (43.5%). They reported no incontinence or fistulas in this group of patients.

Podesta and Urcullo (29) reported on 12 girls with DSD, of which 7 had CAH, who were operated by “perineal mobilization of the UGS” at a mean age of 1.6 years (range 0.4–5.3) and followed for a mean of 7.3 years (3–12). The surgical technique employed in these patients differs from TUM since in the discussion the authors describe “separation of the urethra from the vagina.” There was one introital stenosis requiring revision and one case of mild SUI which resolved with injection of a bulking agent. It should be noted that the diagnosis of the patient who developed SUI is not stated in the paper. This may be relevant since as previously stated the anatomy in patients with other forms of DSD may differ significantly from that of patients with CAH.

It remains to be determined whether the rate of introital stenosis with TUM and a posterior flap will be lower than that reported Bailez et al. (4) and more recently Lesma et al. (28). It is expected that long term results with TUM and a posterior flap will be better since vaginal ischemia and circumferential suture lines are avoided.

We recommend the parents of our patients that an examination under anesthesia should be conducted at the onset of puberty to evaluate the vaginal introitus.

These girls need follow-up till adulthood to evaluate the adequacy of the vagina.

## Conclusion

No case of urinary incontinence has been documented after distal or proximal TUM performed as a primary operation. Although some reports suggest a low incidence of vaginal introital stenosis, no long term results with this technique have yet been published. Concerns about the use of TUM for the correction of virilized genetic females appear unfounded.

## Conflict of Interest Statement

The authors declare that the research was conducted in the absence of any commercial or financial relationships that could be construed as a potential conflict of interest.

## Abbreviations

CAH, congenital adrenal hyperplasia; DSD, disorders of sex development; SUI, stress urinary incontinence; TUM, total urogenital mobilization; UGS, urogenital sinus.
